# Diagnostic elusiveness of pathogenic variants in cases of autosomal recessive diseases

**DOI:** 10.1038/s41431-024-01574-2

**Published:** 2024-03-06

**Authors:** Jörg Schmidtke, Sebastian Koch, Michael Krawczak

**Affiliations:** 1 Hannover Medical School, Carl-Neuberg-Str. 1, 30625 Hannover, Germany; 2 amedes MVZ wagnerstibbe, Georgstrasse 50, 30159 Hannover, Germany; 3 Institute of Medical Informatics and Statistics, Kiel University, Brunswiker Strasse 10, 24105 Kiel, Germany

**Keywords:** Diseases, Genetics

The combination of whole exome sequencing (WES) and subsequent Human Phenotype Ontology-based filtering of the results has become a standard approach in clinical genetics to identifying causative genetic variants in cases of rare human diseases [1]. As recently described by an article in this journal [2], however, the use of this strategy often results in the identification of only a single pathogenic variant for monogenic diseases that are typically bi-allelic. While such an outcome may not definitively resolve the case at hand, it cannot simply be ignored with a view to, for example, taking preventive measures or assessing the risks of relatives. It is intuitively clear that, the rarer the disease, the less likely it is that a single pathogenic allele will be detected in a patient by chance alone.

Here, we present a mathematical approach to evaluating the type of results described above, not least to provide guidance in deciding whether or not further testing (e.g. whole genome sequencing, WGS) should be initiated in a clinical setting. Our aim is to calculate the conditional probability of an ‘elusive’ second allele in cases of overt heterozygosity (genotype G) for a pathogenic variant in gene X, known to be associated with suspected autosomal recessive disease D. This probability depends upon three relevant parameters, namely

- the prevalence π of disease D,
- the analytical sensitivity s of the employed search method, i.e. the probability that a disease-causing variant in gene X is detected by that method,
- the frequency ρ of phenocopies of D, i.e. the prevalence of the patient’s phenotype P among people not affected by D.

Since the presence of a second pathogenic allele is logically equivalent to the unambiguous diagnosis of D, the sought-after conditional probability is

$$P\left(D|G,P\right)=\frac{P\left(D\right)\cdot P\left(G,P|D\right)}{P\left(D\right)\cdot P\left(G,P|D\right)+\left(1-P\left(D\right)\right)\cdot P\left(G,P|{D}^{c}\right)}$$

$$=\frac{\pi \cdot 2s(1-s)}{\pi \cdot 2s\left(1-s\right)+(1-\pi )\cdot \frac{2s\sqrt{\pi }}{1+\sqrt{\pi }}\cdot \rho }$$

$$=\frac{\pi \cdot (1-s)}{\pi \cdot \left(1-s\right)+\frac{(1-\pi )}{1+\sqrt{\pi }}\cdot \sqrt{\pi }\cdot \rho } \sim \frac{\pi \cdot (1-s)}{\pi \cdot \left(1-s\right)+\sqrt{\pi }\cdot \rho }=\frac{\sqrt{\pi }\cdot (1-s)}{\sqrt{\pi }\cdot \left(1-s\right)+\rho }$$

assuming that P and G are conditionally independent in the absence of D (denoted by D^c^), i.e. $P\left(G,P|{D}^{c}\right)=P\left({G|}{D}^{c}\right)\cdot P\left(P|{D}^{c}\right)$, and because

$$P\left(G|{D}^{c}\right)\!=\!\frac{s\cdot 2\sqrt{\pi }(1-\sqrt{\pi })}{{(1-\sqrt{\pi })}^{2}+(1-s)\cdot 2\sqrt{\pi }(1-\sqrt{\pi })+s\cdot 2\sqrt{\pi }(1-\sqrt{\pi })}$$

$$=\frac{s\cdot 2\sqrt{\pi }}{(1-\sqrt{\pi })+(1-s)\cdot 2\sqrt{\pi }+s\cdot 2\sqrt{\pi }}=\frac{2s\sqrt{\pi }}{1+\sqrt{\pi }}$$

**Fig. 1:**
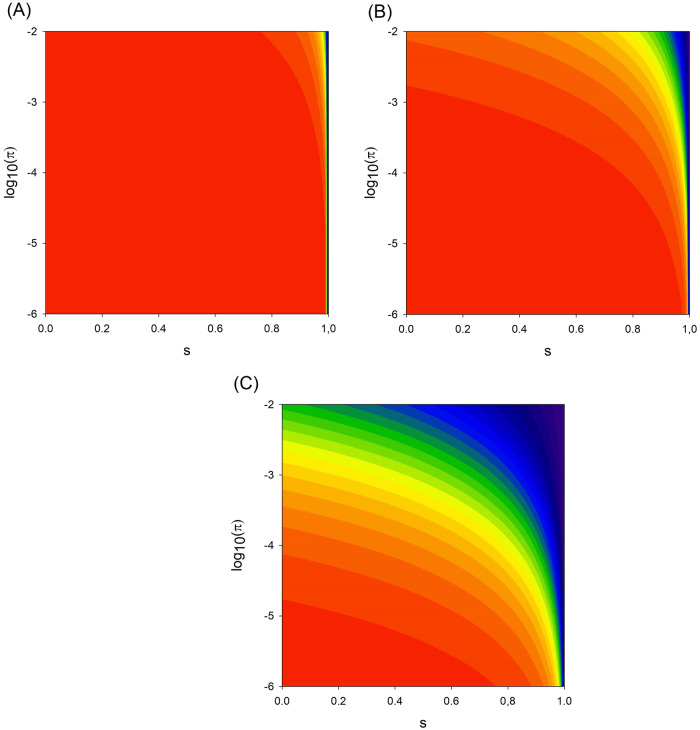
Conditional probability of an ‘elusive’ second allele in cases of overt heterozygosity for a pathogenic variant in a gene associated with an autosomal recessive disease. The probability is color-coded from blue (0) to red (1). π: disease prevalence, s: analytical sensitivity. **A**: prevalence of phenocopies ρ = 0.1 ∙ π, **B**: ρ = π, **C**: ρ = 10 ∙ π.

In practice, reliable prevalence estimates are often rare or even missing, especially if the ethnicity of the individual patient is to be accounted for [3]. Similarly, both the analytical sensitivity of WES and the proportion of phenocopies are mostly unknown for autosomal recessive diseases and their associated genes. Our mathematical derivations imply, however, that even with a high analytical sensitivity and a substantial proportion of phenocopies, a disease with realistic prevalence may still be reliably diagnosed by the detection of a single pathogenic allele, despite the second allele being ‘elusive’ (Fig. 1).

For example, even with an analytical sensitivity as high as 95%, a diagnosis based upon a single variant would still be correct with > 94% probability for a disease with a prevalence of 1:1000 or less and a 10-fold smaller frequency of phenocopies (ρ = 0.1 ∙ π, Fig. 1A). When disease and phenocopies are equally frequent (ρ = π, Fig. 1B), the probability of a correct diagnosis still exceeds 90% for an analytical sensitivity of 90% and a prevalence of 1:10,000 or less. Only if phenocopies clearly dominate (ρ = 10 ∙ π, Fig. 1C) does the probability of a correct diagnosis fall below 50% for that combination of prevalence and analytical sensitivity.

For the molecular genetic diagnosis of a rare autosomal recessive disease, the non-detection of a second disease-causing mutation must not mean the end of it. On the contrary, under realistic assumptions about disease prevalence and analytical sensitivity, the detection of initially only one potentially causative genetic variant in a gene known to be associated with the disease of interest can already mean a sufficiently reliable diagnosis. Regardless of this, however, an attempt should always be made to discover the second ‘elusive’ allele and thus to provide patients and their families with final certainty about their case.
